# Sarcopenia-derived exosomal micro-RNA 16-5p disturbs cardio-repair via a pro-apoptotic mechanism in myocardial infarction in mice

**DOI:** 10.1038/s41598-021-98761-8

**Published:** 2021-09-27

**Authors:** Taiki Hayasaka, Naofumi Takehara, Tatsuya Aonuma, Kohei Kano, Kiwamu Horiuchi, Naoki Nakagawa, Hiroki Tanaka, Jun-ichi Kawabe, Naoyuki Hasebe

**Affiliations:** 1 Division of Cardiology, Nephrology, Pulmonology and Neurology, Department of Internal Medicine, Asahikawa Medical University, 2-1-1-1 Midorigaoka-higashi, Asahikawa, 078-8510 Japan; 2 Division of Tumor Pathology, Department of Pathology, Asahikawa Medical University, Asahikawa, Japan; 3 Division of Integrated Life Science, Department of Biochemistry, Asahikawa Medical University, Asahikawa, Japan

**Keywords:** Myocardial infarction, Macroautophagy, Apoptosis, miRNAs, Experimental models of disease

## Abstract

Sarcopenia is a pathophysiological malfunction induced by skeletal muscle atrophy. Several studies reported an association between sarcopenia-induced cardiac cachexia and poor prognosis in heart disease. However, due to lack of an established animal models, the underlying mechanism of disturbed cardiac repair accompanied with sarcopenia remains poorly understood. Here, we developed a novel sarcopenia-induced cardiac repair disturbance mouse model induced by tail suspension (TS) after cardiac ischemia and reperfusion (I/R). Importantly, we identified a specific exosomal-microRNA marker, miR-16-5p, in the circulating exosomes of I/R-TS mice. Of note, sarcopenia after I/R disturbed cardiac repair and raised the level of circulating-exosomal-miR-16-5p secreting from both the atrophic limbs and heart of TS mice. Likewise, miR-16-5p mimic plasmid disturbed cardiac repair in I/R mice directly. Additionally, in neonatal rat ventricular myocytes (NRVMs) cultured in vitro under hypoxic conditions in the presence of a miR-16-5p mimic, we observed increased apoptosis through p53 and Caspase3 upregulation, and also clarified that autophagosomes were decreased in NRVMs via SESN1 transcript interference-mediated mTOR activation. In conclusion, we show the pro-apoptotic effect of sarcopenia-derived miR-16-5p, which may be behind the exacerbation of myocardial infarction. Therefore, miR-16-5p can be a novel therapeutic target in the context of cardiac repair disturbances in sarcopenia–cachexia.

## Introduction

In the coronary revascularization era, heart failure (HF) after myocardial infarction (MI) remains a fundamental public health problem worldwide. Established coronary intervention prevents the mortality and HF of patients after MI^1^, however, HF incidence in all patients admitted to the hospital with an acute MI still remains as high as 15–35%^2^. Of note, HF in MI patients, often prevents their early mobilization from bed rest. This leads, in turn, to sarcopenia defined as the atrophy of skeletal muscles and the rapid loss of muscle mass and strength^3^. In a full-fledged aged society, we should address the clinical issues that sarcopenia causes to MI patients with HF, leading to cardiac cachexia, often associated with nutritional and metabolic disorders, setting up a vicious circle responsible for progressive cardiac impairment^4–6^.

Thirty-years have progressed since Rosenburg first proposed the definition of "age-related loss of skeletal muscle mass and function" as a sarcopenia^7,8^. It was later re-defined as a decline in skeletal muscle function (either walking speed or grip strength) and was shown to associate with poor outcomes in the context of ischemic heart disease^9^, type II diabetes^10^, cancer^11^, and chronic obstructive pulmonary disease^12^. Even in HF patients with preserved left ventricular function (and without severe heart disease), Bekfani et al*.* reported that low skeletal muscle mass is linked to reduce their cardio-respiratory function and quality of life after HF^13^. Furthermore, it has been reported that elderly patients with large MI or reduced cardiac function prior to hospitalization, who could not achieve an early mobilization post-MI, showed poor outcomes^14,15^. However, though several studies have reported the harmful pathophysiological effect of sarcopenia in patients with cardiac disease accompanied by skeletal muscle loss, the fundamental mechanism behind cardiac cachexia is still unclear. This is probably due to the fact that preclinical models used to study such mechanism could not adequately mimic cardiac cachexia^16–20^. Meanwhile, using genetically engineered dystrophin-deficient mice, where both skeletal muscles and cardiomyocytes are affected, it is hard to study the independent effects of sarcopenia on cardiac impairment^21^. Therefore, to clarify the mechanism of sarcopenia contribution to cardiac cachexia, it is necessary to create a complicated physiologic organ injury model accompanied with multi-organ “non-genetically-induced” sarcopenia and to identify the sarcopenia-derived key molecule.

Here, we established a robust cardiac impairment model in the presence of sarcopenia using non-genetically engineered mice, to evaluate the consequences of sarcopenia with skeletal muscle loss on myocardial ischemia. Furthermore, we identified a specific circulating-exosomal microRNA, miR-16-5p, as a novel regulator accompanied by an organ-linkage in this experimental sarcopenia model. In general, micro-RNAs in a circulating-exosome have been known to modulate the biological process as the protein transcriptional inhibitor encoded by a disease-related gene. Our model is closely mimicking the pathophysiological processes in post-MI patients, therefore, to clarify the mode-of-action of miR-16-5p in sarcopenia may facilitate the development of new post-MI therapeutic strategies, something that is very much needed in an aging society.

## Results

### Skeletal muscle atrophy in I/R mice is induced via sustained tail-suspension (TS)

**Figure 1:**
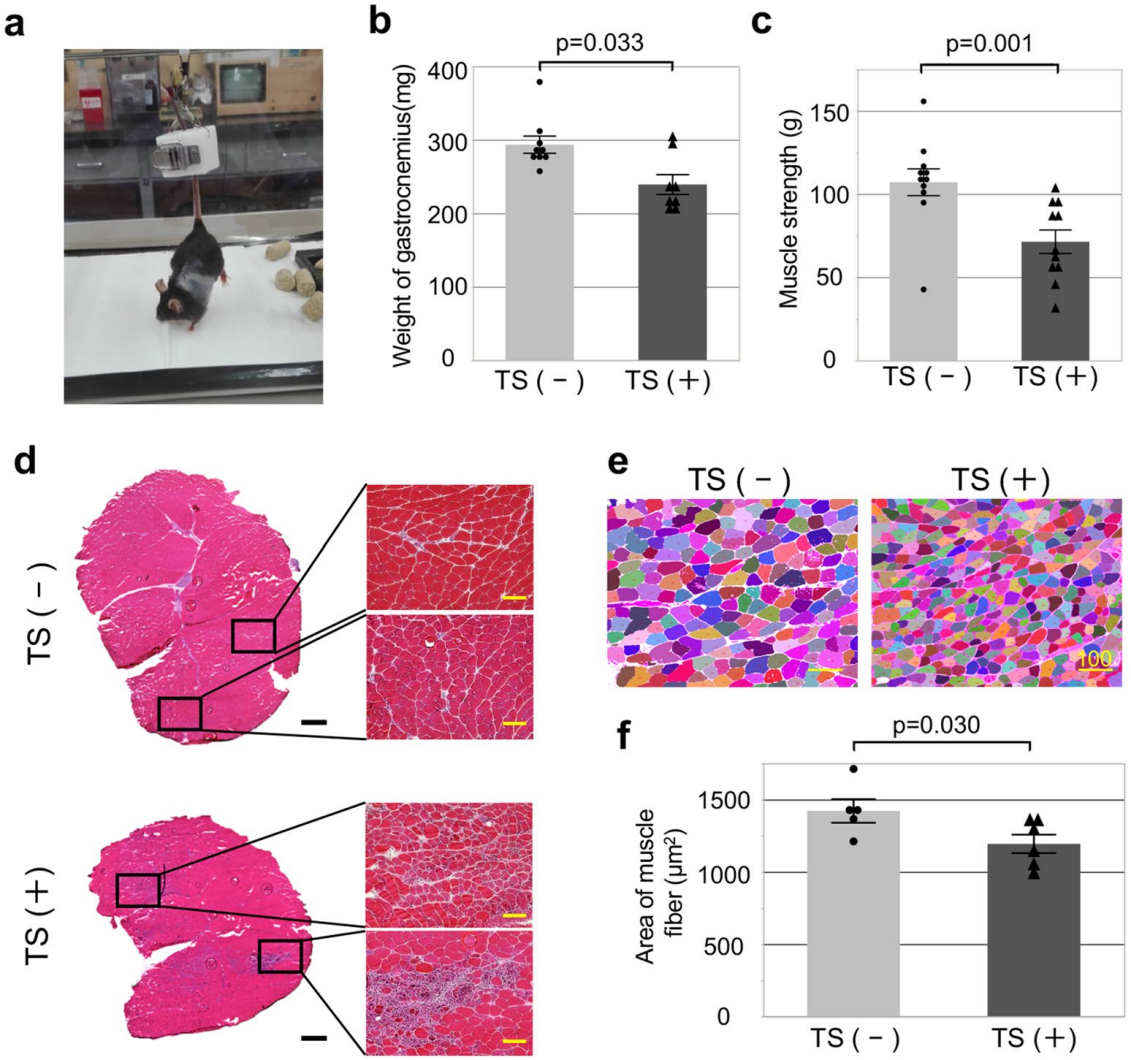
Skeletal muscle atrophy due to sustained tail-suspension after I/R. (**a**) Experimental sarcopenia was induced via using the modified Morey’s tail-suspension (TS) model. Analysis of the weight of the gastrocnemius (**b**) and of the limb strength (**c**) in TS (+) (n = 8) and TS (−) (n = 9) mice. Left bar; TS (−), Right bar; TS (+). (**d**) An evident reduction of the myofiber cross-sectional area (CSA) was observed in TS (+) mice. Histological findings of the gastrocnemius of TS (+) (lower) and TS (−) (upper) mice. Left side; cross-section of the gastrocnemius; scale bar = 500 μm. Right side; enlarged figure of the myofibers; scale bar = 100 μm. (**e**) Automatic detection of the CSA of myofibers using the BZ-X Analyzer software (Keyence. Co.). Scale bar = 100 μm. (**f**) Analysis of the CSA of the myofibers. Left bar; TS (−), Right bar; TS (+).

Experimental sarcopenia was induced in I/R mice using the modified Morey’s tail-suspension (TS) model from days 1 to 8 after I/R (Fig. 1a)^22^. To assess the effect of modified tail-suspension on the skeletal muscles, I/R mice subjected or not to TS [TS (+), or TS (−), respectively] were randomly assigned to two groups and compared. One week after TS, the muscle weight [gastrocnemius; 293.5 ± 11.7 vs. 239.3 ± 13.5 mg in TS (−) vs TS (+) mice, respectively; p = 0.033] and the muscle strength [107.2 ± 8.1 vs 71.5 ± 7.1 g in TS (−) vs. TS (+) mice, respectively; p = 0.001] were significantly decreased in TS (+) mice compared to in TS (−) mice (Fig. 1b, c). Additionally, as per the histological analysis, the gastrocnemius muscle of TS (+) mice showed atrophied muscle fibers and increased interstitial tissues with localized inflammatory cell infiltration (Fig. 1d). Of note, comparing the fiber cross-sectional area (CSA) present per a unit area in the two groups revealed an evident reduction in TS (+) mice compared to TS (−) mice [TS (−) vs. TS (+); 1421.1 ± 81.4 vs. 1193.4 ± 63 0.7 µm^2^, respectively; p = 0.030; Fig. 1e, f]. In line with these results, the liver and lungs weight of TS (+) mice were slightly decreased after modified tail-suspension compared to those in TS (−) mice, and the body weight was comparable between the two groups (Supplementary Figure 1).

### Experimental sarcopenia disturbs cardiac repair after I/R

**Figure 2:**
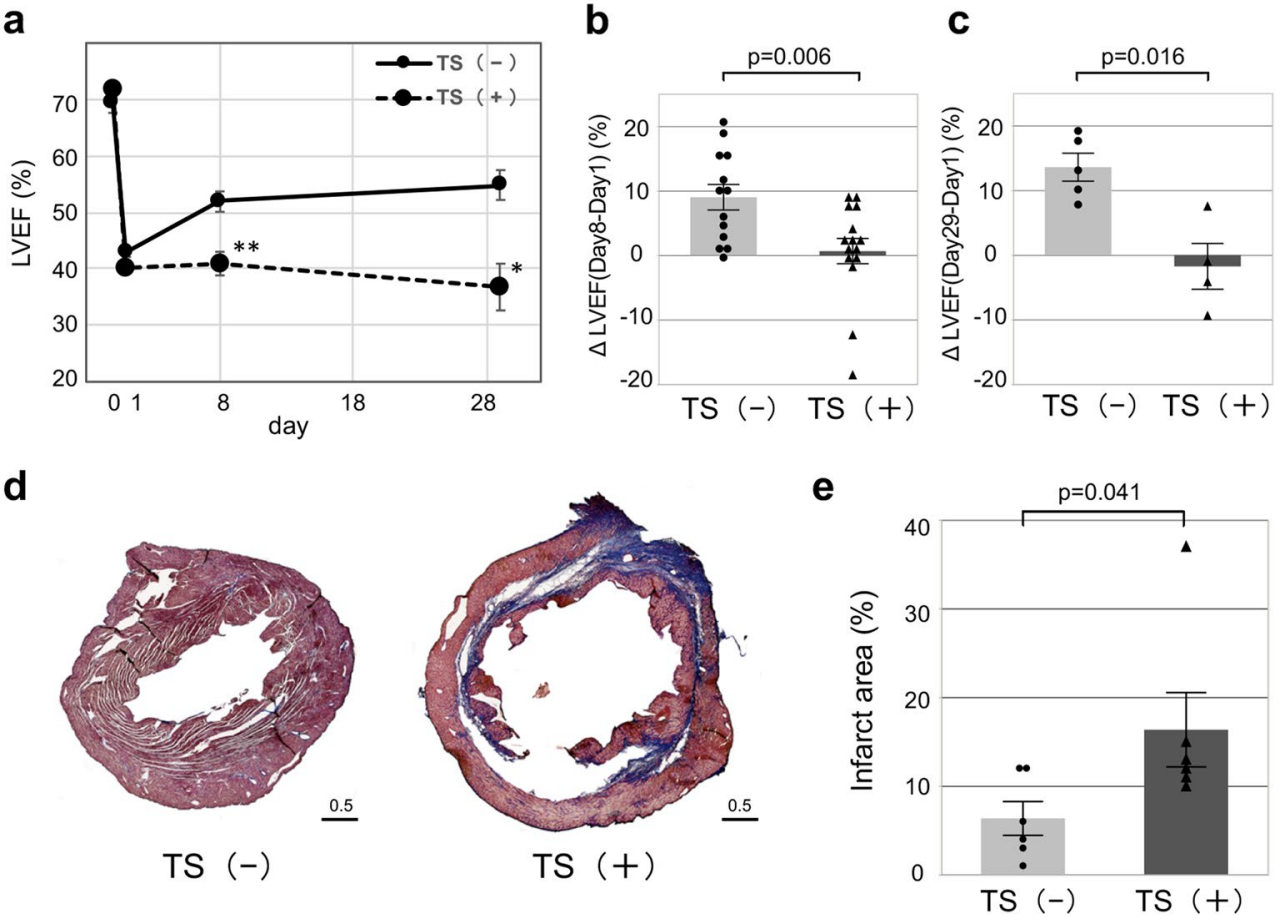
Experimental sarcopenia impairs cardiac after I/R. (**a**) Absolute changes in the LVEF on days 1, 8 in TS (+) (n = 15) and TS (−) (n = 13; 2 mouse early death), days 29 in TS (+) (n = 5) and TS (−) (n = 4), ΔLVEF (**b**, Day 8–Day 1, **c** Day 29—Day 1) in the 2 groups of mice. Left bar; TS (−), Right bar; TS (+). *p < 0.05, **p < 0.001 vs. TS (+). (**d**) Representative images of cardiac tissue sections (short axis view; day 28 post-MI) stained with Masson’s Trichrome. The fibrotic areas are stained blue. Left side; TS (−). Right side; TS (+). Scale bar = 0.5 mm. (**e**) Quantitative analysis of the percentage of the fibrotic area as a function of the total LV area (n = 6 per group). Left bar; TS (−), Right bar; TS (+).

To assess the impact of experimental sarcopenia on the cardiac function after I/R, the left ventricular ejection fraction (LVEF) of 42 I/R mice (short term; 8 days n = 30, long term; 29 days n = 12) in the two groups was evaluated using echocardiography. Two mice in TS (−) (short term) were experienced unexpected early death, and 3 mice [TS (+) = 1, TS (−) = 2] (long term) had unsuitable images for analysis due to chest operation scar. Importantly, in the current I/R model used (45 min transient coronary ligation), the LV dysfunction is expected to partially improve 1 week after coronary reperfusion. Nevertheless, 8 days after I/R, LV dysfunction was not ameliorated in I/R-TS (+) mice, in contrast to that in I/R-TS (−) mice [LVEF (%), TS (+) = 40.3 ± 0.8 to 40.9 ± 2.1%, TS (−) = 42.9 ± 0.8 to 51.9 ± 1.8%; ΔLVEF = TS (+) vs. TS (−) = 0.6 ± 2.0 vs. 9.0 ± 2.0, p = 0.006, Fig. 2a, b]. Further, we continued to assess the cardiac function of I/R mice for one month with and without experimentally-induced sarcopenia. The improved LV function of I/R-TS (−) mice at day 8 was maintained at day 29 (LVEF = 54.8 ± 2.6%), while LV function of I/R-TS (+) mice was continuously disturbed from day 1 to day 29 (LVEF = 38.8 ± 4.1%); of note, there was a significant difference between the two groups [ΔLVEF (Day 29–Day 1) (%) = − 1.8 ± 3.5 vs. 13.6 ± 2.0 in TS (+) vs. TS (−) mice, respectively; p = 0.016, Fig. 2c].

We also evaluated the infarct size in I/R-mice using Masson’s Trichrome staining at day 29 to clarify the effect of the modified TS method on persistent myocardial damage. An area of intense staining in the myocardium of I/R-TS (+) mice was larger emphasized than that of I/R-TS (−) mice (Fig. 2d). Further, the infarct size in I/R-TS (+) mice was significantly greater than that of I/R-TS (−) mice (16.3 ± 4.2 vs. 6.3 ± 1.9%, respectively; p = 0.041, Fig. 2e).

### Exosomal micro-RNAs in the context of experimental sarcopenia after I/R

**Figure 3:**
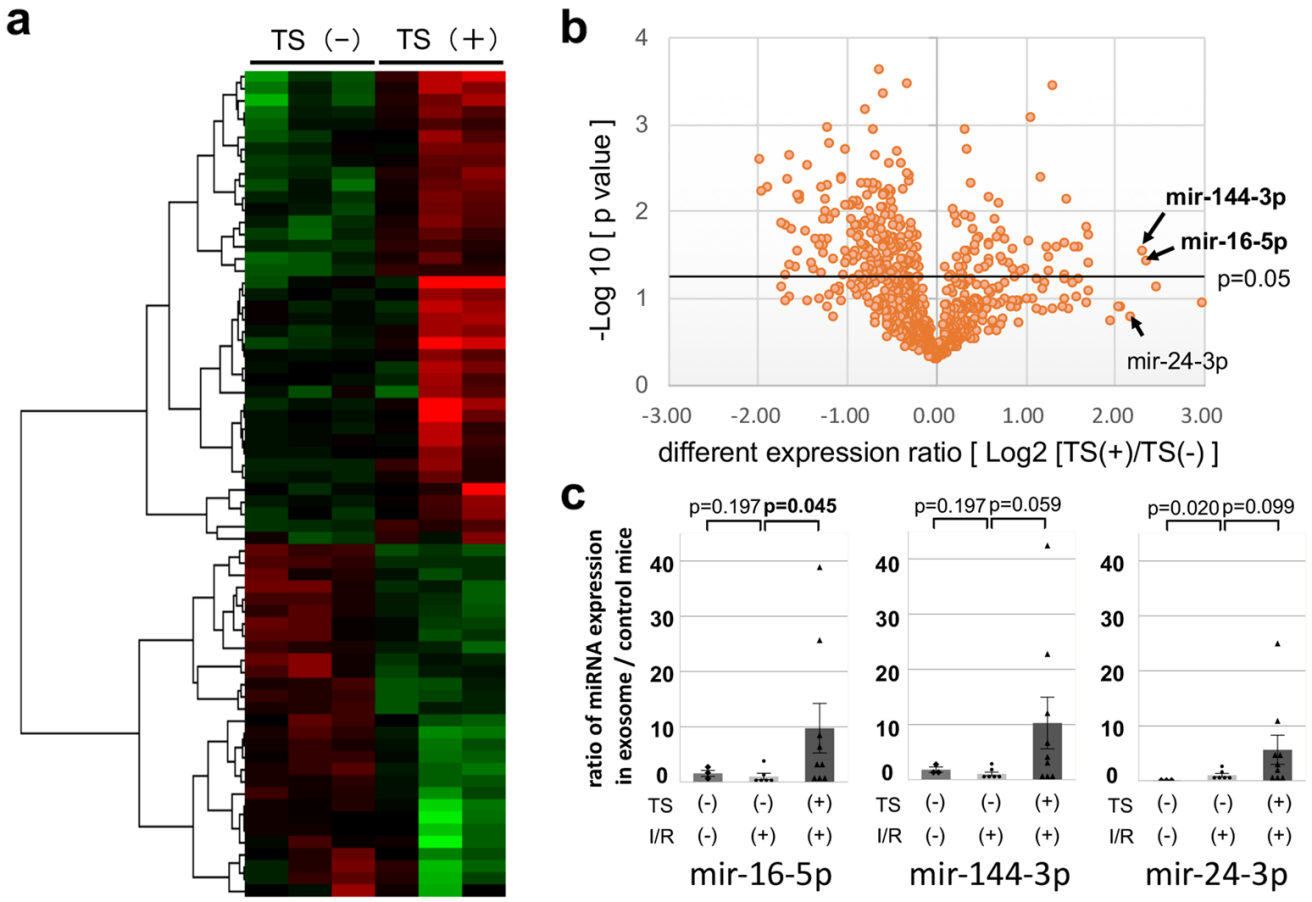
Specific exosomal-micro-RNAs in sarcopenic mice after I/R. (**a**) Comprehensive cluster analysis of miRNAs expression in the exosomes from I/R mice (both groups) showing the 68 differentially expressed miRNAs (fold change > 2.0). Green; down regulated miRNAs. Red; upregulated miRNAs. Left 3 lines; TS (−) mice. Right 3 lines; TS (+) mice. (**b**) Volcano plot analysis of 944 miRNAs. The horizontal lines show the p-values with respect to the differential expression analysis in the two groups. Two candidate miRNAs (upregulated) with a high differential expression ratio were plotted over the horizontal line. (**c**) Quantitative (q) RT-PCR analysis of the expression of miR-16-5p, miR-144-3p, and miR-24-3p in the two groups of I/R mice [I/R (+) TS (+); n = 9, I/R (+) TS (−); n = 6, I/R (−) TS (−); n = 3]. Left bar; I/R (−) TS (−). Center bar; I/R (+) TS (−). Right bar; I/R (+) TS (+).

Next, circulating exosomes were extracted from the whole blood of both I/R mice after the release of TS (day 8) and the total RNA of exosomes was purified and subjected to micro-RNA array analysis; 3D-Gene global miRNA microarray mouse chips encompassing all mouse miRNAs available on the Sanger miRBase were used in the two groups (n = 3 mice per group) to identify a specific exosomal miRNA which exerted the cardio-repair disturbance in I/R-TS (+) mice. A comprehensive cluster analysis of the expression of miRNAs in the exosomes from both groups of I/R mice showed that the cardio-repair disturbance was associated with the differential expression of 68 miRNAs [fold change > ± 2.0 (log2 > ± 1.0), Fig. 3a].

Notably, the expression of 42 miRNAs (among the 68 differentially expressed) was significantly up or down regulated in I/R-TS (+) mice (p < 0.05, Supplementary Table 1). Further, we identified two upregulated candidate micro-RNAs, miR-16-5-p and miR-144-3p, showing with a > 4-fold change (log_2_ > 2.0) as shown in the Volcano plot analysis (Fig. 3b). Finally, we selected these two micro-RNA candidates (miR-16-5-p, miR-144-3p), and miR-24-3p (well known as cancer and I/R heart related miRNA, which had a high expression ratio but no statistical variance in this microarray analysis; validation control) for subsequent validation via qRT-PCR. The expression level of miR-16-5p and miR-144-3p was not significantly different between mouse with and without I/R condition, however, we confirmed that the expression of exosomal miR-16-5p was clearly upregulated in response to the tail-suspension after I/R in this validation study [expression ratio; I/R (+)-TS (−) vs. I/R (+)-TS (+) = 1.0 ± 0.6 vs. 9.7 ± 4.5%, respectively; p = 0.045, Fig. 3c]. Based on our results, we selected a miR-16-5p as the candidate miRNA most likely associated with the cardio-repair disturbance in I/R mice with sarcopenia.

### A miR-16-5-p mimic promotes hypoxia-induced apoptosis in NRVMs

**Figure 4:**
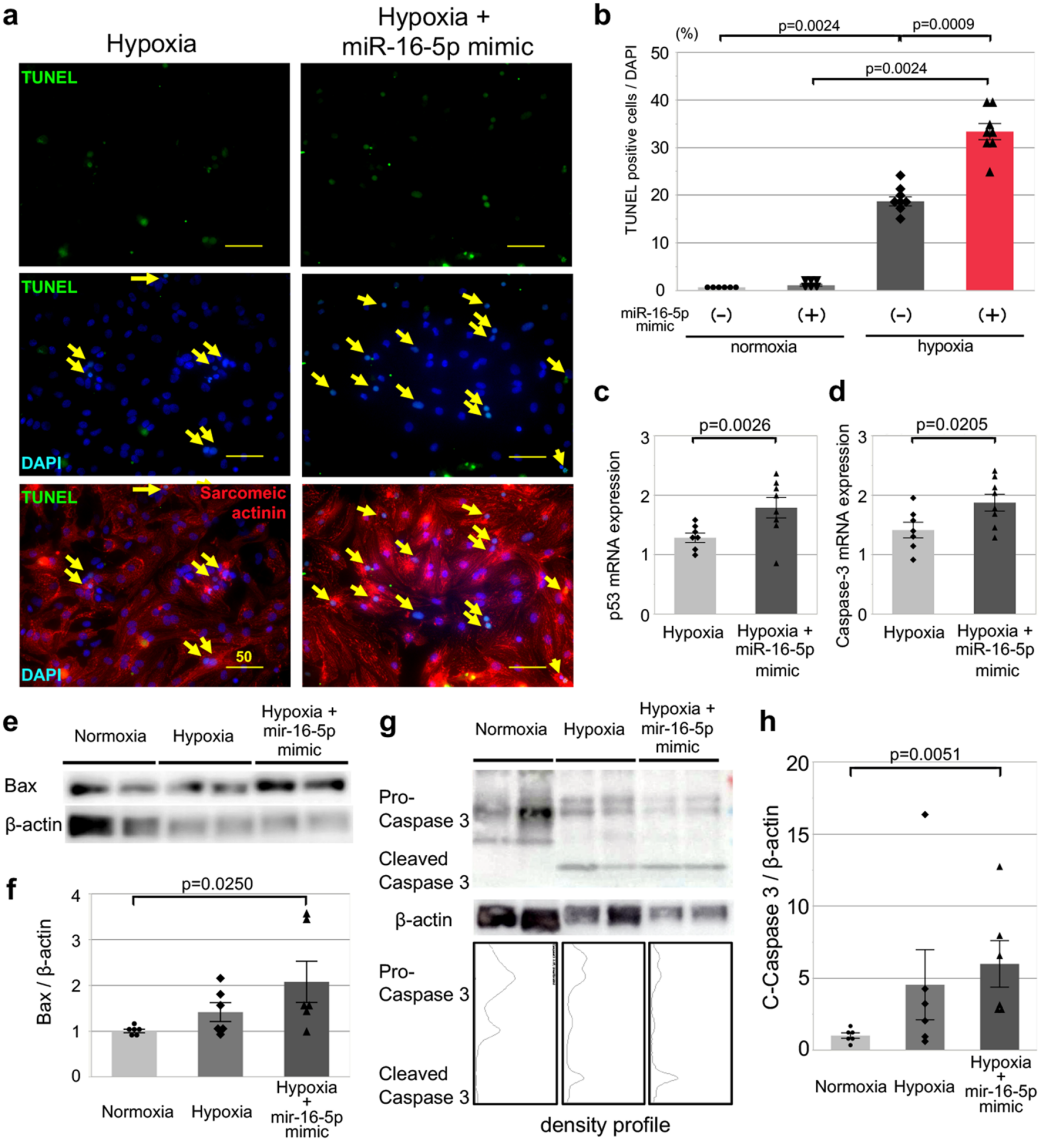
Enhanced hypoxia-induced apoptosis after miR-16-5-p mimic-transfection. (**a**) Representative images of TUNEL-positive cells [green]; nuclei were stained with 4ʹ, 6-diamidino-2-phenylindole (DAPI) [blue]. NRVMs were labeled with an antibody against cardiac α-sarcomeric actinin (SA) [white]. Left side; NRVMs under hypoxia. Right side; NRVMs under hypoxia with miR-16-5p mimic transfection. Upper panel; TUNEL-positive cell. Middle panel; merged image of TUNEL, DAPI. Lower panel; merged image of TUNEL, DAPI, and SA image. Yellow arrows; representative TUNEL-positive cells. Scale bar = 50 μm. (**b**) The ratio of TUNEL-positive apoptotic NRVMs (normoxia; n = 6, hypoxia; n = 8). Left side; normoxia with (left bar) and without (right bar) miR-16-5p mimic transfection. Right side; hypoxia with (left bar) and without (right bar) miR-16-5p mimic transfection. The percentage of apoptotic NRVMs in all NRVMs, as evaluated by the TUNEL assay. Quantitative (q) RT-PCR analysis of the mRNA expression in NRVMs. The expression of *p53* (**c**) and *Caspase-3* (**d**) in NRVMs (All; n = 7) transfected with miR-16-5p mimic was also evaluated. Left bar; hypoxia. Right bar; hypoxia with miR-16-5p mimic transfection. Western blotting (**e**,**g**) and analysis of the protein expression of Bax (**f**), and cleaved caspase-3 (**h**) in NRVMs (All; n = 6) transfected with miR-16-5p mimic was also evaluated. Left bar; normoxia. Center bar; hypoxia. Right bar; hypoxia with miR-16-5p mimic transfection.

Circulating exosomal-miR-16-5p was significantly upregulated only until day 8 after myocardial ischemia in I/R-TS (+) mice; however, these mice exhibited persistent cardio-repair disturbance until day 29. Therefore, we hypothesize that early systemic exposure to miR-16-5p after myocardial ischemia directly induced persistent cardiomyocyte death. To test this hypothesis, we assessed apoptosis in the context of in vitro normoxic and hypoxic cultures of neonatal rat ventricular myocytes (NRVMs) transfecting with (or without) an miR-16-5p mimic plasmid. The transferase dUTP nick end labeling (TUNEL) assay revealed no differences in the apoptosis of NRVMs transfected with or without the miR-16-5p mimic under normoxic conditions. On the other hand, the apoptosis induced in NRVMs under hypoxic condition for 48 h was significantly enhanced after transfection with the miR-16-5p mimic (normoxia vs. hypoxia vs. hypoxia + miR-16-5-p mimic = 0.6 ± 0.1 vs. 18.7 ± 1.0 vs. 33.3 ± 1.7%, respectively; normoxia vs. hypoxia; p = 0.0024, hypoxia vs. hypoxia + miR-16-5-p mimic; p = 0.0009, Fig. 4a, b).

Furthermore, qRT-PCR analysis demonstrated that transfection with the miR-16-5p resulted in the upregulation of *p53* (hypoxia vs. hypoxia + miR-16-5-p mimic = 1.2 ± 0.1 vs. 1.9 ± 0.1%, p = 0.001, Fig. 4c) and *caspase-3* (hypoxia vs. hypoxia + miR-16-5-p mimic = 1.4 ± 0.3 vs. 1.9 ± 0.4%, p = 0.021, Fig. 4d) in NRVMs under hypoxic condition. In addition, western blotting analysis revealed that transfection with the miR-16-5p mimic under hypoxic condition resulted in the protein upregulation of Bax (normoxia vs. hypoxia + miR-16-5-p mimic = 1.00 ± 0.04 vs. 2.07 ± 0.45, p = 0.0250, Fig. 4e, f) and cleaved caspase-3 following the modification of caspase 3 profile (normoxia vs. hypoxia + miR-16-5-p mimic = 1.00 ± 0.19 vs. 5.98 ± 1.62, p = 0.0051, Fig. 4g, h) compared with normoxic condition. Therefore, collectively, these results suggest that miR-16-5p modulates the apoptosis of NRVMs under hypoxic conditions via the p53-induced classical pathway.

### miR-16-5p inhibits autophagy in NRVMs via the transcriptional interference of SESN1

**Figure 5:**
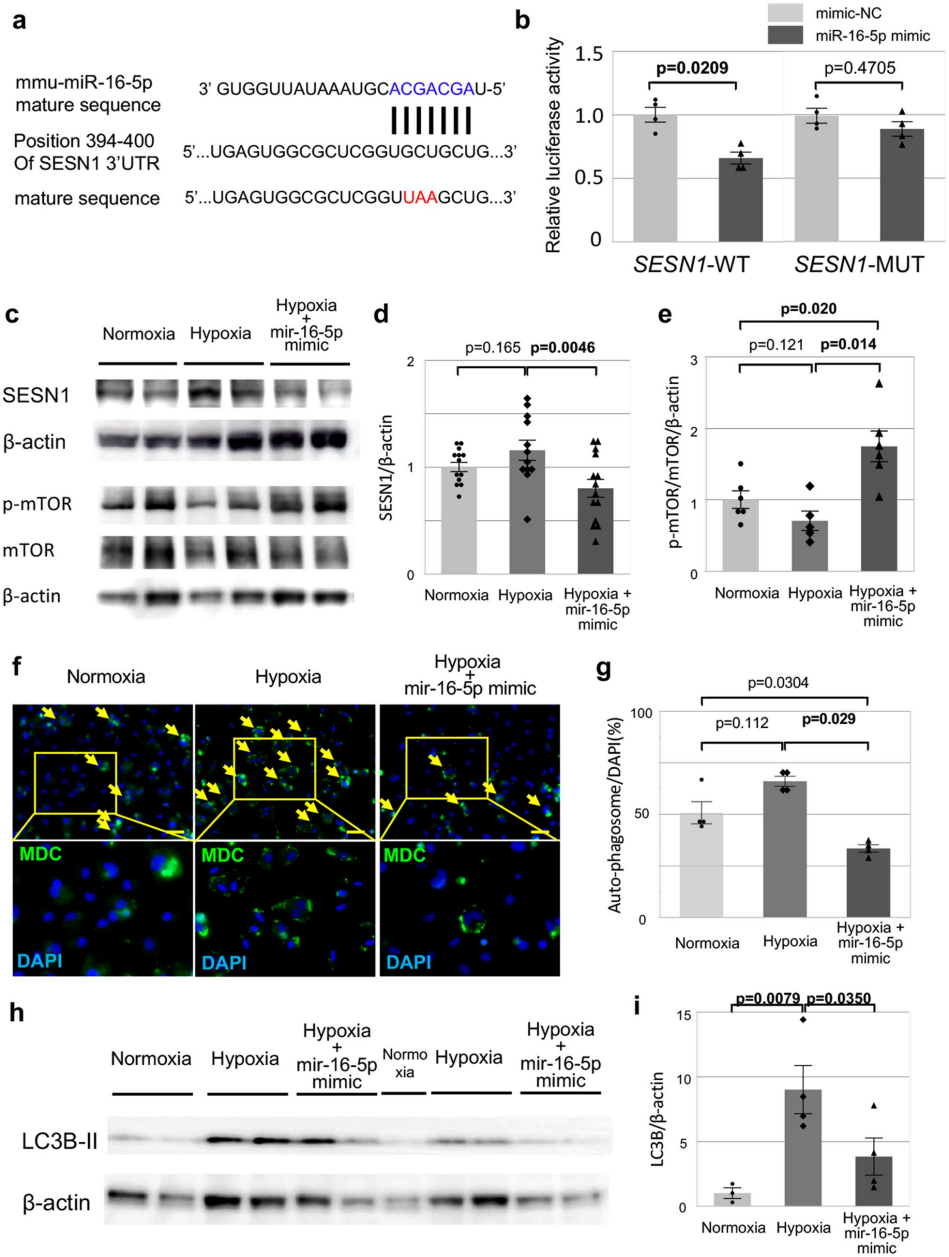
Suppression of the autophagy in NRVMs via the miR-16-5p-mediated transcriptional suppression of *SESN1.* (**a**) Target/binding site of SESN1 and miR-16-5p, and *Sesn1* mutation sequence. (**b**) Luciferase assay; SESN1 contains a target gene of mir-16-5p. Left side; *Sesn1*-3ʹUTR-wild type, Right side; *Sesn1*-3ʹUTR-mutant, Gray bar; miR-16-5p mimic (n = 4), Light gray bar; miR-mimic control (n = 4), (**c**) Representative blots of SESN1, phosphorylated-mTOR, mTOR, and β-actin protein expression in NRVMs. Left side; normoxia. Center; hypoxia. Right side; hypoxia with miR-16-5p mimic transfection. (**d**) Fold change of SESN1 activation in NRVMs under hypoxia with (n = 14) and without miR-16-5p mimic transfection (n = 12); the baseline refers to NRVMs under normoxia (n = 13). (**e**) Fold change of mTOR-phosphorylation in NRVMs under hypoxia with (n = 6) and without miR-16-5p mimic transfection (n = 5); the baseline refers to NRVMs under normoxia (n = 6). Left bar; normoxia. Center bar; hypoxia. Right bar; hypoxia with miR-16-5p transfection. (**f**) Representative images of autophagosomes in NRVMs (All; n = 4) [monodansylcadaverine (MDC); green]; the nuclei were stained with DAPI [blue]. Left side; normoxia. Center; hypoxia. Right side; hypoxia with miR-16-5p transfection. The upper panel is a low magnitude image. The lower panel is an expanded image of autophagosomes and DAPI-positive cells. Yellow arrow; representative autophagosome-positive cells. Scale bar = 50 μm. (**g**) The ratio of autophagosome positive NRVMs (All; n = 4). Left bar; normoxia. Center bar; hypoxia. Right bar; hypoxia with miR-16-5p transfection. The percentage of autophagosome positive NRVMs in all NRVMs is represented. Western blotting (**h**) and analysis of the protein expression of LC3B-II (**i**) in NRVMs (normo n = 3, the others; n = 4; respectively) transfected with miR-16-5p mimic was also evaluated. Left bar; normoxia. Center bar; hypoxia. Right bar; hypoxia with miR-16-5p mimic transfection.

To clarify the mechanism underlying the pro-apoptotic effects of miR-16-5p, we searched for miR-16-5p target genes using the microRNA database (miRDB; http://mirdb.org/mirdb). Initially, 186 genes with target scores over 90 points were selected among 1175 potential miR-16-5p targets. Among them, we searched the anti-apoptotic gene which is associated with cell death of myoblast or cardiomyocyte stimulated by the p53 gene. As a result, we focused on the SESN1/2 gene which is known as an anti-apoptotic/pro-autophagy regulator protein in the cellular response to DNA damage and oxidative stress^23^. Next, we searched the miRbase (miRBase; http://www.mirbase.org), and found that the seed sequence of miR-16-5p corresponded directly with nucleotides 394–400 in the 3ʹ UTR of mouse *Sesn1* mRNA (Fig. 5a). To confirm whether miR-16-5p directly targets the 3ʹ UTR of SESN1, a dual-luciferase reporter assay was performed in SNL cell (mouse embryonic fibroblast cell line). The recombinant reporter vectors (SESN1-WT and -MUT) were co-transfected with miR-16-5p mimic or mimic-normal control (mimic-NC). The luciferase activity of the wild-type group (SESN1-3ʹ UTR-WT) was significantly decreased after transfection with miR-16-5p mimic (mimic-NC vs. miR-16-5p mimic; 1.000 ± 0.058 vs. 0.659 ± 0.047%, respectively; p = 0.0209, Fig. 5b), whereas no significant difference was observed in the mutant group (SESN1-3ʹ UTR-MT). Therefore, we focused on this particular target hereafter, using the miR-16-5p mimic in the in vitro/in vivo system.

Interestingly, hypoxic conditions tended to increase the SESN1 protein levels in NRVMs, although not significantly (normoxia vs. hypoxia; 1.00 ± 0.04 vs. 1.16 ± 0.09%, respectively; p = 0.165, Fig. 5c,d). Meanwhile, transfection with the miR-16-5p mimic significantly downregulated the SESN1 protein levels in NRVMs under hypoxia conditions (hypoxia vs. hypoxia + miR-16-5-p mimic; 1.16 ± 0.09 vs. 0.80 ± 0.08%, respectively; 0.0046, Fig. 5c,d). As SESN1 is associated with the target of rapamycin (TOR) kinase, we further investigated the effect of miR-16-5p on the phosphorylation of mTOR in NRVMs under hypoxic conditions. Expectedly, the phosphorylation of mTOR in NRVMs was greatly enhanced by the miR-16-5p mimic under hypoxia conditions (hypoxia vs. hypoxia + miR-16-5-p mimic; 0.70 ± 0.13 vs. 1.74 ± 0.22%, respectively; p = 0.014, Fig. 5c,e).

We then investigated the effect of miR-16-5p on autophagy. The presence of green-fluorescent monodansylcadaverine (MDC; a fluorescent marker for autophagic vacuoles) exhibiting autophagosomes on NRVMs was observed in the steady-state under normoxic condition (Fig. 5f,g). These autophagosomes tended to increase under hypoxia condition, further, western blotting analysis revealed the inducible autophagy defined as the high expression of LC3B-II protein under hypoxia condition (normoxia vs. hypoxia = 1.00 ± 0.41 vs. 8.99 ± 1.86, p = 0.0079, Fig. 5h,i). Importantly, transfection of the miR-16-5p mimic under hypoxia significantly decreased the frequency of MDC-positive autophagosomes in NRVMs (hypoxia vs. hypoxia + miR-16-5-p mimic; 65.9 ± 2.4 vs. 33.4 ± 1.8%, respectively; p = 0.029, Fig. 5f,g) and also inhibited the level of LCBII-protein of NRVMs (hypoxia vs. hypoxia + miR-16-5p mimic = 8.99 ± 1.86 vs. 3.82 ± 1.44, p = 0.0350, Fig. 5h,i). Taken together, these results suggest that miR-16-5p results in mTOR signaling upregulation of via the transcriptional interference of *SESN1*, thus negatively regulating autophagy in NRVMs under hypoxia.

### Circulating miR-16-5p is derived from the atrophic limbs and heart of sarcopenic mice and directly interferes with the restoration of LV dysfunction in I/R mice

**Figure 6:**
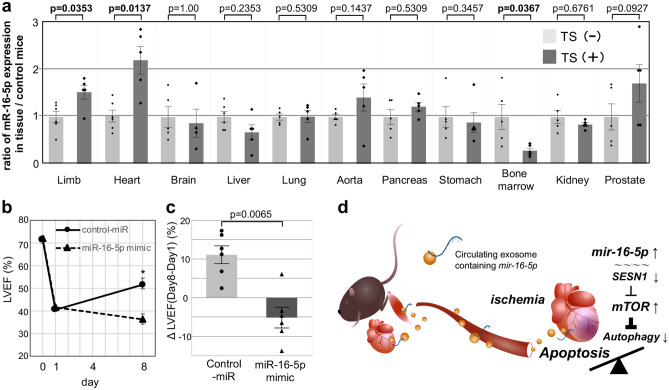
Circulating miR-16-5p is derived from the atrophic-limbs and hearts of sarcopenic mice and interferes with restoration of LV dysfunction in I/R mice. **a,** qRT-PCR analysis of the expression of miR-16-5p in the brain, liver, limbs, heart, lung, aorta, pancreas, stomach, bone marrow, kidney, and prostate of mice subjected [TS (+)] or not [TS (−)] tail-suspension (all; n = 5). The ratio of miR-16-5p expression in TS (+) mice was calculated compared that in TS (−) mice (standardized to the expression level of 1). Light gray bar; TS (−), Dark gray bar; TS (+). (**b**) Absolute changes in the LVEF on days 8 in miR-16-5p mimic (n = 6) and control-miR mimic (n = 6), *p < 0.05. (**c**) ΔLVEF (Day 8–Day 1) in the 2 groups of mice. Left bar; control-miR mimic, Right bar; miR-16-5p mimic. (**d**) Schematic image of the proposed mechanism. Cardio-repair disturbance in the context of sarcopenia is mediated by exosomal-miR-16-5p secreted from the atrophic limbs and hearts. miR-16-5p directly interferes with the transcription of *SESN1*, and then activates mTOR signaling, which in turn induces cell apoptosis.

As a result of the miR-16-5p mimic in vitro study, an exosomal miR-16-5p might affect the cardio-repair disturbance due to induce the pro-apoptotic effect to ischemic cardiomyocytes in sarcopenia mice after I/R. Accordingly, the following question was raised; from which tissue did the exosomal miR-16-5p original from in mice? To address this query, we performed organ profiling using qRT-PCR to assess the exosomal miR-16-5p source in the context of our in vivo model by determining the expression of miR-16-5p in different tissues (brain, heart, limb, liver, lung, aorta, pancreas, stomach, bone marrow, kidney, and prostate) of sarcopenic mice without I/R induction (Fig. 6a). In general, the TS method is recognized as a model of depression, however, no difference in the expression of miR-16-5p was observed in the brain of sarcopenic mice. Similarly, no difference in the expression levels of miR-16-5p was also observed in the other tissues (liver, lung, aorta, pancreas, stomach, kidney, prostate) of sarcopenic mice in the two groups*,* in contrast, the expression of miR-16-5p in the bone marrow of TS (+) mice was decreased versus that in TS (−) mice (0.28 ± 0.05 vs. 1.00 ± 0.26, respectively; p = 0.0367). Meanwhile, the expression of miR-16-5p in the atrophic limbs of TS (+) mice 7 days after a tail-suspension was significantly increased versus that in TS (−) mice (1.0 ± 0.12 vs. 1.53 ± 0.15, respectively; p = 0.0353). In addition, the expression of miR-16-5p in the heart of TS (+) mice was also significantly increased versus that in TS (−) mice, even in the absence of I/R (1.02 ± 0.13 vs. 2.21 ± 0.30, respectively; p = 0.0137).

LV dysfunction of I/R-TS (+) mice was not ameliorated after limb unloading. This might be associated with circulating-exosomal miR-16-5p released from the hind-limb and heart of sarcopenia mice (TS mice). To investigate the direct impact of upregulated circulating-exosomal miR-16-5p on the cardio-repair disturbance in I/R-TS (+) mouse, we administered the miR-16-5p mimic or control-miR mimic plasmid to I/R-TS (−) mice intravenously along with atelocollagen^24^. Similar to that observed in the current mouse I/R model, the LV dysfunction of I/R-TS (−) mice after coronary reperfusion was partially improved at 1 week even after injection of control-miR mimic plasmid. However, 7 days after injection of miR-16-5p mimic plasmid, LV dysfunction was not ameliorated in I/R-TS (−) mice, an observation that was in contrast to that in I/R-TS (−) mice injected with the control-miR mimic [LVEF (%), control-miR mimic = 40.5 ± 1.2 to 51.5 ± 3.0%, miR-16-5p mimic = 41.5 ± 1.1 to 36.3 ± 2.4%, Day 8, p = 0.0104, Fig. 6b; ΔLVEF = control-miR mimic vs. miR-16-5p mimic = 11.1 ± 2.3 vs. − 5.2 ± 2.7, p = 0.0065, Fig. 6c]. It means that the circulating miR-16-5p disturbed cardiac repair in I/R-TS (−) mice directly.

## Discussion

Here, we developed a robust I/R-based cardiac impairment mice model in the presence of sarcopenia. We, further, successfully employed our novel model to identify a cardiotoxic exosomal micro-RNA and characterized miR-16-5p, which acts by decreasing autophagy and promoting cardiomyocyte apoptosis, as a pivotal player in cardiac impairment. The prevention of sarcopenia after myocardial infarction remains a global health care issue, therefore, we should not also let it forgetting as a pathophysiological cardio-repair intervention against an exosomal micro-RNA interference.

Micro-RNAs are known interference regulators of gene transcription, and play an important role in the autocrine and/or paracrine repair of injured tissues. For instance, several micro-RNAs, including miR-21^25^, miR-29^26^, and miR-25^27^, were defined as biomarkers in the context of cardiac hypertrophy, MI, and HF^28^. Additionally, MiR-1^29^, miR-133^30^, and miR-208^31^ were reported to be upregulated in the context of embryonic heart development from cardiac crescent to fetal heart as autocrine regulators^32^. These micro-RNAs, known as cardiac regulators, have been mainly studied concerning their associations with the cardiac cycle, regeneration, and cardiomyocyte proliferation^32^. However, much less is known in the context of pathophysiological conditions such as HF and/or cardiac cachexia. Of note, a few “cardio-regulated” micro-RNAs suggested as biomarkers of HF may be mobilized from other organs in patients with heart disease. In the present study, we established a cardio-repair disturbance model mimicking cardiac cachexia based on limb unloading-induced sarcopenia after myocardial ischemia/reperfusion. Previously, Hughes et al. reported that miR-31 in the atrophic skeletal muscles of aged rats was transiently upregulated after mechanical limb unloading, injuring the skeletal muscles in an autocrine manner^33^. Here, via multiorgan profiling in sarcopenic mice we suggested that miR-16-5p upregulation in the atrophic limbs was streamed as circulating-exosomal micro-RNAs, which reached and subsequently impacted the heart. Based on micro-RNA mimic in vitro studies, sarcopenia-induced circulating-exosomal miR-16-5p may be closely associated with deterioration of an injured heart and generate a state of “cardiac cachexia”. Interestingly, although there were no apparent increased preload signs in the lungs and liver of sarcopenic mice miR-16-5p was also elevated in the heart after limb unloading without I/R. Probably, the induction of sarcopenia not only leads to skeletal muscle atrophy, to a prodromal state of “cardiac cachexia” like with downregulated miR-16-5p expression in bone marrow, which may, in turn, also upregulate cardiac miR-16-5p expression. Taken together, our results collectively suggest that miR-16-5p is a novel “cardio-regulated” micro-RNA whose expression is induced in the context of sarcopenia and is responsible for “cardiac cachexia” (Fig. 6d).

miR-16 belongs to the micro-RNA-15 family (consisting of miR-15a/b, miR-16-1/2, miR-195 and miR-497), and is a well-known tumor suppressor, highly expressed in several cancers, including prostate cancer, lung cancer, and chronic lymphocytic lymphoma. Additionally, it was previously reported that the members of the micro-RNA-15 family are important for the regulation of the differentiation of cardiomyocytes and skeletal muscle cells. Porrello et al. described that the inhibition of the expression of miR-195 at an early postnatal stage decreased the proliferation of myocytes not only in embryo- but also in postnatal hearts and exacerbated to the left ventricular systolic function in adult mice after MI^34^. miR-16-5p, the one this study focuses on has the homologous sequence “AGCAGC”, found in all of the micro-RNA-15 family members, and regulates the transcription of genes related to cell proliferation, regeneration, and death. Recently, Cai et al. reported that miR-16-5p directly targets the *SESN1* gene impacting the proliferation and apoptosis of myoblasts, and consequently the differentiation of skeletal muscles^35^. Additionally, Li et al. described the role of SESN1/2 in doxorubicin cardiotoxicity using SESN1/2 double-knockout mice^36^; however, the regulator of SESN1 in the context of cardiac injury has not yet been determined in their model. In the current study, when we transfected NRVMs with a miR-16-5p mimic-encoding plasmid, a pro-apoptotic effect was observed under hypoxic conditions in vitro; of note, the same was not true under normoxic conditions. In general, excessive oxidative stress leads to apoptosis through increased p53 expression; this said, p53 may also stimulate cytoprotective pathway to maintain cell homeostasis, regulating autophagy by mTOR dephosphorylation, via SESN1 upregulation^37^. Because miR-16-5p directly inhibits the transcription of SESN1, miR-16-5p has a pro-apoptotic effect, leading to an imbalance of apoptosis and autophagy under oxidative stress (Fig. 6d). Interestingly, in the current study, miR-16-5p could not promote the apoptosis of NRVMs under normoxic conditions, and the cardio-repair disturbance in the context of the I/R mouse model used was not observed in the absence of sarcopenia. This suggests that exosomal miR-16-5p, in the absence of oxidative stress, does not impact apoptosis and autophagy in cardiomyocytes; only exosomal miR-16-5p accompanied with a sarcopenia–cachexia intervention promotes the deterioration of myocardial injury via a pathophysiological mal-adaptation. As well known in previous study, autophagy usually plays a pivotal role in controlling cell viability, providing the necessary nutrients during starvation. Therefore, the mobilization of exosomal miR-16-5p may inhibit the autophagy-based self-repair of injured organs in a sarcopenia–cachexia environment. That is one reason explaining why a conventional clinical approach to treat myocardial ischemia would not revert cardio-repair disturbances after MI in the context of sarcopenia. Hence whenever early adaptation interventions (e.g., exercise intervention) after MI are impossible, due to aging or severe HF, therapeutic approaches targeting the miR-16-5p-SESN1 axis may be ideal alternatives, in the future.

Certain limitations were noted in the current study. For instance, in this study, we did not adopt a genetic model mouse to validate the loss of function of miR-16-5p. However, the deletion of the miR-15/16 clusters in mice has been shown to result in a shorter lifespan due to acute myeloid leukemia^38^; therefore, we believed that miR-15/16 KO mice would not be a suitable model for the analysis of physiologic organ injury to assess the outcome of myocardial infarction. Additionally, we were unable to fully elucidate about the upregulation of cardiac miR-16-5p of TS (+) mouse in detail. In this study, we confirmed no acceleration of a circulating exosomal-miR-16-5p and cardiac (endogenous) miR-16-5p (Supplementary Figure S2) after I/R without tail suspension. Meanwhile, TS (+) mouse without I/R had no heart failure and less alteration of general condition by limb unloading (Supplementary Figure S1). Therefore, after myocardial ischemia, a sarcopenia induced by limb unloading may lead to a constitutional specific alteration as a "cardiac cachexia”, which associated with circulating exosomal-miR-16-5p acting as an "external" secretory cardiac regulated micro RNA—as well as cardiac endogenous miR-16-5p. Direct interactions between limb unloading and endogenous cardiac miR-16-5p will be investigated further in subsequent studies.

In conclusion, we show that the induction of sarcopenia after I/R injury promotes cardiac repair disturbance together with the increased expression of circulating-exosomal-miR-16-5p. We further demonstrate that miR-16-5p promotes apoptosis in cardiomyocytes via SESN1, hence, the miR-16-5p-SESN1 axis should be considered as a potential target for new treatment strategies for heart disease patients that are unable to undergo exercise intervention-based early adaptation due to aging or severe HF.

## Methods

### Ethics statement

All procedures including animal studies were conducted following the guidelines for the Care and Use of Laboratory Animals of the Ministry of Education, Culture, Sports, Science and Technology, Japan. The animal experiments were approved by the Institutional Animal Care and Use Committee (IACUC)/ethics committee of the Asahikawa Medical University (protocol number 19101). All experiments and methods were carried out in compliance with relevant regulations and Animal Research: Reporting of In Vivo Experiments (ARRIVE) guidelines.

### Mouse model of I/R, sarcopenia, and miR mimic plasmid injection

8–10-week-old C57BL/6 male mice were anesthetized and ventilated with 3% isoflurane after intubation. The left anterior (coronary) descending artery (LAD) was occluded directly under the left atrium using monofilament nylon 8-0 sutures (Ethicon, Somerville, NJ, USA) for 45 min; then, the occlusion was released (I/R procedure). The occlusion time of the LAD was designed as 45 min to enable for the partial recovery of the contraction of the left ventricle. Additionally, for sarcopenia induction, mice were tail-suspended for 7 days in individual cages. This TS protocol employs a pulley block that maintains forelimb activity and does not interfere with food or fluid intake. Moreover, a horizontal balance must be achieved to avoid applying a steep angle to the animal’s body, thus, permitting normal weight bearing on the forelimbs, as has been described previously^39,40^. I/R mice were injected with miR-16-5p mimic plasmid (sense-5ʹ-uagcagcacguaaauauuggcg-3ʹ, antisense-5ʹ-cgccaauauuuacgugcugcuauu-3ʹ) or negative control-miR mimic plasmid (sense-5ʹ-auccgcgcgauaguacguaTT-3ʹ, antisense-5ʹ-uacguacuaucgcgcggauTT-3ʹ) (Koken CO, Tokyo, Japan) at day 1 and 4 after coronary reperfusion. In brief, 4 nmol/body of miR-16-5p mimic, or control-miR mimic was mixed with 100 µL Dulbecco's Phosphate Buffered Saline (DPBS). Atelocollagen (AteloGene^®^, Koken CO) was diluted in an equal volume of DPBS to attain a final concentration of 0.1% by pipetting up and down for 20 times, and rotating for 15 min at 4 °C. After these two solutions were mixed together by pipetting up and down for 20 times, the mixture (200 μL for each mouse) was then delivered into each mice via tail vein with an insulin syringe (27G, 1 mL). LV function of I/R mice was evaluated at day 7 after miR-mimic plasmid injection by echocardiography.

### Histological analysis (hematoxylin and eosin, and Masson’s trichrome staining)

Gastrocnemius muscle samples were harvested from TS mice (n = 10, respectively) 8 days after tail-suspension. Hearts were harvested from TS mice (n = 6, respectively) 21 days after tail-suspension. All samples were fixed in 4% paraformaldehyde (PFA), followed by treatment with sucrose solution. Frozen sections (7 µm for the hearts and 10 µm for skeletal muscles) were then obtained. Three gastrocnemius muscle samples [TS (+) = 2, TS (−) = 1] that were unsuitable for analysis due to damage during storage or freezing were treated as outliers. Hematoxylin and eosin staining^38^ were conducted on gastrocnemius sections to analyze the myofiber size. Images were acquired using a fluorescence microscope (BZ-X710; Keyence, Osaka, Japan), and the area of myofibers was measured using the BZ-X Analyzer software (BZ-H3A/H3C ver.1.3.1.1 Keyence. Co.). The heart sections were subjected to Masson’s Trichrome staining^38^ to evaluate fibrosis; the fibrotic area was measured using the Image-J software (ver.1.53a; National Institutes of Health, Bethesda, MD, USA. http://imagej.nih.gov/ij). Additionally, the infarct size was calculated as the percentage of fibrosis area within the total LV area.

### Echocardiography

30 (short term) and 12 (long term) mice were divided into groups using a random number table after the surgery and transthoracic echocardiography was performed to evaluate heart function before and 1, 8, and 29 days after the I/R procedure, using the Vevo 660 system (VisualSonics, Toronto, Canada). B-mode images of hearts were recorded from the parasternal short and long-axis view. The end-systolic and end-diastolic left intraventricular areas (basal, mid and apical) in the short-axis view were measured. The LV ejection fraction (LVEF) was calculated using the following formula: V = (area mid-ventricular + area apical +  area basal) × h/3, where h = ventricular length.

The ejection fraction was calculated for both methods using the formula: EF = (EDV − ESV)/EDV × 100. The assessment was performed in a blinded manner.

### Extraction of micro-RNAs from circulating-exosomes

Total exosomes were isolated from the serum using the Total Exosome Isolation Reagent Kit (Thermo Fisher Scientific, Waltham, MA, USA). Briefly, the collected blood was centrifuged at 2000*g* for 10 min to obtain the serum. The serum was further centrifuged at 2000*g* for 30 min to completely remove the cells and debris, and 40 µL of reagent was added to 200 µL of the obtained serum and incubated at 4 °C for 30 min. After incubation, samples were centrifuged at 1000*g* for 10 min, and the exosomes remaining at the bottom of the tube were lysed in a resuspension buffer. Total RNA was extracted from the resulting exosome-containing samples using the Total Exosome RNA and Protein Isolation Kit (Thermo Fisher Scientific) and reversely-transcribed into cDNA using the TaqMan Micro-RNA Reverse Transcription Kit, as per the manufacturers’ instructions.

### miRNA microarray analysis

For the micro-RNA microarray analysis, total RNA samples were extracted from circulating-exosome of TS (−) and TS (+) mice (n = 3), and their quality was checked using the Bioanalyzer system (Agilent, Santa Clara, CA, USA). miRNA expression was analyzed using the 3D-Gene miRNA Oligo chip and 3D-Gene miRNA labeling kit. Briefly, half volumes of labeled RNAs were hybridized onto a 3D-Gene miRNA Oligo chip designed to detect 2565 miRNA sequences; the annotation and oligonucleotide sequences of the probes were conformed to the miRbase. Hybridization signals were scanned using the 3D-Gene Scanner3000 and processed using the 3D-Gene Extraction software (All materials; Toray, Tokyo, Japan). The detected signals for each gene were normalized using the global normalization method. The candidate micro-RNAs assigned as differentially expressed with an adjusted p-value < 0.05 (one-sided *t* test) were narrowed down among genes differentially expressed over four-fold between the 2 groups.

### Quantitative RT-PCR (qRT-PCR)

Total RNAs from cultured NRVMs, and circulating exosomes, gastrocnemius muscle, liver, brain, heart, lung, aorta, pancreas, stomach, bone marrow, kidney, prostate samples [TS (−) and TS (+) mice] were extracted using the RNeasy Mini kit (Qiagen, Valencia, CA, USA)^41^. Total RNAs from cultured NRVMs were reversely-transcribed into cDNAs using SuperScript-III Reverse Transcriptase (Invitrogen, Carlsbad, CA, USA). To assess the expression of tissue-derived miR-16-5p, exosomal miR-16-5p, -miR-144-3p, -miR-24-3p, and -U6 as an endogenous control, real-time TaqMan reverse transcription PCR was performed using the TaqMan micro-RNA Assay kit (Applied Biosystems, Foster City, CA, USA) following the manufacturer’s protocol. Quantitative (q)-RT-PCR for the rat *p53*, *caspase-3*, and *GAPDH* in the context of NRVMs was performed using the TaqMan Gene expression Master Mix (Applied Biosystems) on a Light Cycler^®^ 96 System (Roche, Basel Switzerland).

### Luciferase reporter-based miR-16-5p targeting assay

The *Sesn1* 3ʹ-untranslated region (3ʹ-UTR) with the miR-16-5p binding site was cloned into the pmirGLO Dual-Luciferase miR Target Expression Vector (E1130, Promega). The following oligonucleotide pairs were designed, annealed, and ligated into the pmirGLO Vector to generate WT and mutant luciferase constructs: WT forward primer, 5ʹ-AAACTAGCGGCCGCTAGTTGAGTGGCGCTCGGTGCTGCTGT-3ʹ, WT reverse primer, 5ʹ-CTAGACAGCAGCACCGAGCGCCACTCAACTAGCGGCCGCTAGTTT-3ʹ, mutant forward primer, 5ʹ-AAACTAGCGGCCGCTAGTTGAGTGGCGCTCGGTTAAGCTGT-3ʹ, and mutant reverse primer, 5ʹ-CTAGACAGCTTAACCGAGCGCCACTCAACTAGCG GCCGCTAGTTT-3ʹ. SNL cells seeded in 96-well plates were transfected with 0.1 μg of a luciferase plasmid along with either 50 nM of miR mimic control or miR-16-5p mimic. At 48 h after transfection, the transfected SNL cells were used to evaluate luciferase activities in Firefly and Renilla buffers measured with the Dual-Glo Luciferase Assay System (E2920, Promega) with the GloMax^®^ Navigator Microplate Luminometer (Promega).

### microRNA mimic transfection in the context of NRVMs under hypoxic conditions

NRVMs were obtained from neonatal (1-day old) SD rat hearts following the manufacturer’s protocol^38^. NRVMs were allowed to reach 50% confluency, to determine the conditions that enable the best visualization of NRVM apoptosis and autophagosomes. Six days after seeding to allow NRVM maturation, the culture media was replaced with serum-free medium (DMEM/F12, Thermo Fisher Scientific K.K. Japan), and then, cells were cultured for 2 days in a hypoxic environment containing 5% CO_2_, 1% O_2_, and 94% NO_2_ in an incubator. To investigate the effect of miR-16-5p in the context of NRVMs, a miR-16-5p mimic [50 nM; miRVana miRNA mimics (Ambion)] was transfected using Lipofectamine RNAiMAX Reagent (Thermo Fisher Scientific) for 2 days in a hypoxic environment. The TUNEL assay and the detection of autophagosomes were performed after hypoxic cultivation for 2 days.

### TUNEL analysis

For the terminal deoxynucleotidyl TUNEL assay, cells were fixed with 2% paraformaldehyde for 10 min at room temperature. After permeabilization with phosphate-buffered saline containing 0.1% Triton-X and 0.1% sodium citrate for 2 min at 4 °C, cells were incubated with fluorescein isothiocyanate (FITC)-conjugated TUNEL reaction mixture (In situ Cell Death Detection kit, Roche Diagnostics, Indianapolis, IN, USA) for 60 min at 37 °C. Samples were then stained with 4ʹ,6-diamidino-2-phenylindole to label the nuclei and visualized under an epifluorescence microscope; the BZ-X Analyzer (Keyence. Co.). TUNEL-positive cells were counted in at least six randomly selected microscopic fields under a 10 × objective.

### Western blotting

NRVMs were homogenized on ice in radioimmunoprecipitation assay lysis buffer (Santa Cruz Biotechnology, Dallas, TX) containing the protease inhibitors leupeptin (5 µg/mL), aprotinin (2 µg/mL), and phenylmethylsulphonyl fluoride (PMSF; 1 mM). Lysates were centrifuged at 12,000*g* for 20 min at 4 °C and the supernatants were collected. The protein concentrations were determined, and aliquots containing 50 mg of protein were separated by electrophoresis on 4–12% Blot Bis–Tris Gels (Invitrogen Japan) in a NuPAGE MOPS SDS Running Buffer system (Invitrogen Japan), and sequentially transferred onto nitrocellulose membranes, according to the manufacturer’s instructions. Proteins were blotted using iBind Western Systems (Thermo Fisher Scientific) and detected by chemiluminescence (#34096, Thermo Fisher Scientific; #NEL113001EA, PerkinElmer, Inc., Waltham, MA, USA; ImageQuant LAS500, GE Healthcare UK Ltd., Buckinghamshire, England). The following primary antibodies used in this study were: anti-Bax (#14796), anti-caspase3 (#14220)*,* anti-Cleaved caspase3 (#9661; All of them; Cell Signaling Technology)*,* anti-SESN1 (ab134091; Abcam, Cambridge, MA, USA), anti-p-mTOR (S2448), anti-mTOR (7C10), anti-LC3B (#2775), and anti-β-actin (All of them; Cell Signaling Technology).

### Detection of autophagosomes

Autophagosomes were detected using the Cell Meter Autophagy Assay Kit *Green Fluorescence* (AAT Bioquest, Inc., Sunnyvale, CA, USA), after NRVMs were subjected to 1% hypoxia 48 h with or without miR-16-5p mimic transfection. Briefly, the NRVMs were stained with the Autophagy Green™ working solution and incubated at 37 °C for 30 min; then the nuclei were stained with Hoechst 33342 (Lonza, Walkersville, MD, US) for 10 min. Cells were washed three times and examined under a fluorescence microscope, the BZ-X Analyzer. The ratio of autophagosome-positive cells was calculated as per the number of NRVMs containing autophagosomes, versus that of nuclei in at least six randomly selected microscopic fields under a 20 × objective.

### Statistical analysis

Experimental data are presented as the mean ± the standard error (SE). The number of samples (n) is disclosed in the respective figure legends. Significance was determined using the Wilcoxon analysis or the student t-test in case of normally distributed*.* A p-value < 0.05 was considered significant. Data were analyzed using JMP14.0 (JMP, Tokyo, SAS).

## Supplementary Information

Supplementary Information.

## Data Availability

All of the data supporting this study’s findings are available within the article and its Supplementary Materials.
