# Supplementary material for: Sarcopenia-derived exosomal micro-RNA 16-5p disturbs cardio-repair via a pro-apoptotic mechanism in myocardial infarction in mice
Source: Sci Rep. 2021 Sep 27;11:19163. doi: 10.1038/s41598-021-98761-8 (PMC8476608; doi:10.1038/s41598-021-98761-8)

Sarcopenia-derived exosomal micro-RNA 16-5p disturbes cardio-repair via a pro-apoptotic mechanism in myocardial infarction in mice.

Taiki Hayasaka[1], Naofumi Takehara[1*], Tatsuya Aonuma[1], Kohei Kano[1], Kiwamu Horiuchi[1], Naoki Nakagawa[1], Hiroki Tanaka[2], Jun-ichi Kawabe[3], and Naoyuki Hasebe[1]

1 Department of Internal Medicine, Division of Cardiology, Nephrology, Pulmonology and Neurology, Asahikawa Medical University, Asahikawa, Japan
2 Department of Pathology, Division of Tumor Pathology, Asahikawa Medical University, Asahikawa, Japan
3 Department of Biochemistry, Division of Integrated Life Science, Asahikawa Medical University, Asahikawa, Japan

*Address correspondence to:
Naofumi Takehara
2-1-1-1 Midorigaoka-higashi, Asahikawa 078-8510, Japan
Tel: +81-166-68-2442; Fax: +81-166-68-2449
E-mail address: takenao1@mac.com

Supplementary Figure Legends

Supplementary Figure S1. The weights of the whole body, lungs, and liver of experimental mice.

Analysis of the weight of the whole body (A), liver (B), and lungs (C) of TS (+) (n = 6) and TS (-) (n = 4) mice. Left bar; TS (-). Right bar; TS (+).

Supplementary Figure S2. The ratio of miR-16-5p expression in mice heart with or without I/R.

Analysis of the expression of the miR-16-5p in heart of I/R (+) (n = 4) and I/R(-) (n = 6) mice. Left bar; I/R(-). Right bar; I/R(+).

Supplementary Table 1. The 42 differentially expressed miRNAs with statistical variance.

3D-Gene global miRNA microarray mouse chips based on the Sanger miRBase in the two groups (n = 3). A comprehensive cluster analysis of the expression of miRNAs in the exosomes from the two groups of I/R mice showed that the characteristics of TS (+) depended on the regulation of 68 miRNAs (fold change > 2.0); 42 of them, were found to be differentially expressed with statistical variance miRNAs ($p < 0.05$).

Supplementary Information 1. Gels and blots of the images Fig. 4e, g

Gels and Western blots of Figure 4e and Figure 4g. Figure 4e; Bax and β-actin.
Figure 4g; Pro-/Cleaved-Caspase and β-actin.

Supplementary Information 2. Gels and blots of the images Fig. 5c

Gels and Western blots of Figure 5c. Left side Figure 5c; SESN1and β-actin.
Right side Figure 5c; p-mTOR, mTOR, and β-actin.

Supplementary Information 3. Gels and blots of the images Fig. 5h

Gels and Western blots of Figure 5h. Figure 5h; LC3B-II and β-actin.

Supplemental Figure 1

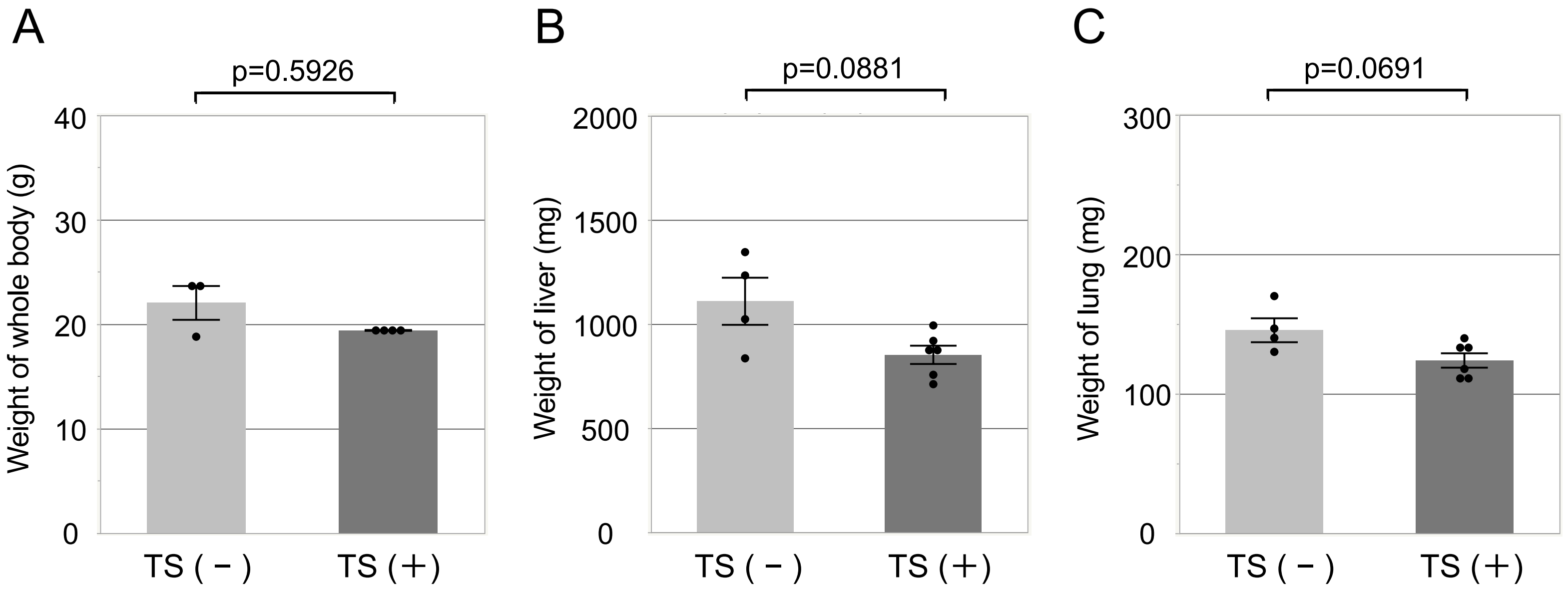

Supplemental Figure S2

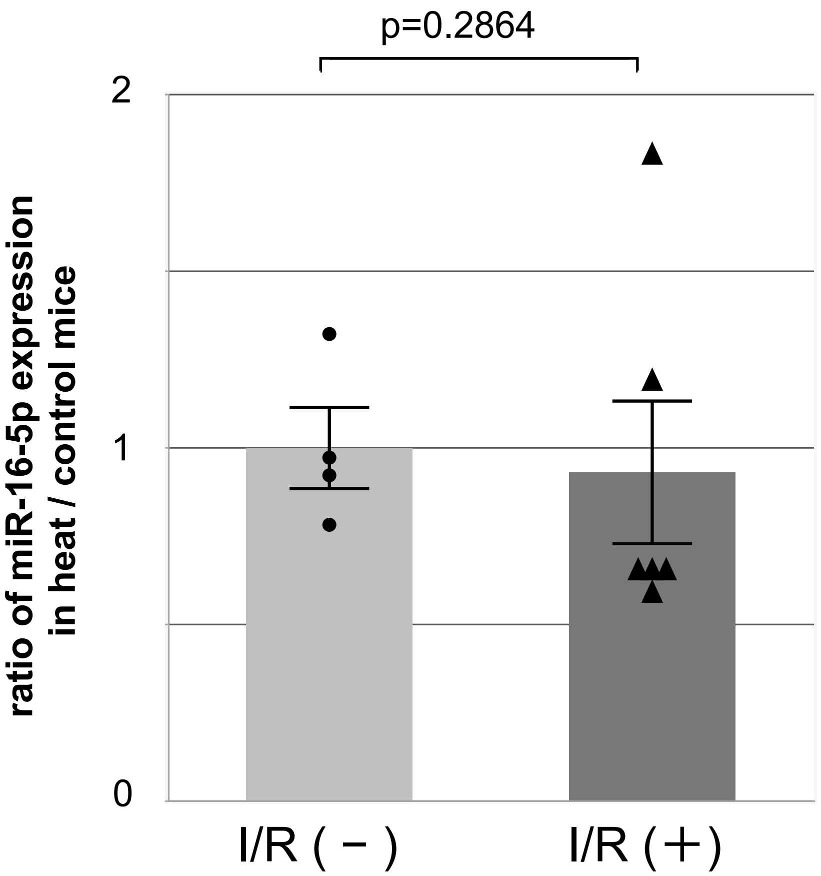

Supplemental Table 1

Name	ID	Average		ratio TS(+) / TS (-)	LOG2ratio TS(+) / TS (-)	p-value(F)	p-value(t)
		TS (-)	TS (+)				
mmu-miR-144-3p	MIMAT0000156	31	159	5.10	2.35	0.04	0.038
mmu-miR-16-5p	MIMAT0000527	29	145	5.01	2.32	0.08	0.026
mmu-miR-30c-5p	MIMAT0000514	15	44	3.04	1.60	0.12	0.026
mmu-miR-30d-5p	MIMAT0000515	15	31	2.06	1.04	0.55	0.022
mmu-miR-709	MIMAT0003499	54	131	2.42	1.27	0.06	0.048
mmu-miR-2136	MIMAT0011212	42	100	2.36	1.24	0.14	0.025
mmu-miR-15a-5p	MIMAT0000526	10	32	3.30	1.72	0.03	0.019
mmu-miR-15b-5p	MIMAT0000124	21	67	3.24	1.70	0.20	0.015
mmu-miR-365-1-5p	MIMAT0017077	73	187	2.55	1.35	0.04	0.026
mmu-miR-365-2-5p	MIMAT0017179	18	44	2.39	1.26	0.14	0.034
mmu-miR-30b-5p	MIMAT0000130	12	34	2.96	1.57	0.09	0.027
mmu-miR-6969-5p	MIMAT0027840	23	63	2.77	1.47	0.25	0.007
mmu-miR-6922-3p	MIMAT0027745	9	19	2.08	1.05	0.09	0.001
mmu-miR-7077-5p	MIMAT0028060	29	78	2.72	1.44	0.20	0.024
mmu-miR-5621-5p	MIMAT0022369	114	282	2.48	1.31	0.51	0.000
mmu-miR-6999-5p	MIMAT0027900	32	104	3.28	1.71	0.02	0.040
mmu-miR-7081-5p	MIMAT0028068	290	580	2.00	1.00	0.01	0.046
mmu-miR-3572-5p	MIMAT0022986	136	307	2.26	1.18	0.09	0.004
mmu-miR-7662-5p	MIMAT0029830	50	16	0.32	-1.63	0.08	0.002
mmu-miR-615-5p	MIMAT0004837	108	47	0.44	-1.20	0.00	0.002
mmu-miR-1900	MIMAT0007870	18	8	0.45	-1.15	0.12	0.036
mmu-miR-5116	MIMAT0020624	303	95	0.31	-1.67	0.12	0.014
mmu-miR-6415	MIMAT0025169	21	8	0.41	-1.28	0.53	0.005
mmu-let-7f-1-3p	MIMAT0004623	18	7	0.42	-1.26	0.22	0.012
mmu-miR-455-3p	MIMAT0003742	14	6	0.40	-1.31	0.19	0.024
mmu-miR-7653-3p	MIMAT0029813	60	26	0.43	-1.21	0.27	0.021
mmu-miR-1892	MIMAT0007871	134	67	0.49	-1.02	0.98	0.008
mmu-miR-1947-3p	MIMAT0017343	11	4	0.34	-1.54	0.02	0.029
mmu-miR-3091-5p	MIMAT0014903	10	4	0.39	-1.37	0.05	0.047
mmu-miR-221-3p	MIMAT0000669	14	6	0.43	-1.21	0.62	0.005
mmu-miR-378a-5p	MIMAT0000742	8	3	0.43	-1.21	0.90	0.001
mmu-miR-7033-3p	MIMAT0027971	9	4	0.48	-1.05	0.71	0.004
mmu-miR-292a-5p	MIMAT0000369	13	5	0.42	-1.26	0.42	0.031
mmu-miR-290a-5p	MIMAT0000366	52	18	0.34	-1.55	0.87	0.006
mmu-miR-1901	MIMAT0007880	54	25	0.46	-1.11	0.72	0.010
mmu-miR-138-5p	MIMAT0000150	16	7	0.42	-1.24	0.96	0.010
mmu-miR-1893	MIMAT0007879	764	264	0.35	-1.53	0.48	0.007
mmu-miR-8109	MIMAT0031415	27	13	0.48	-1.05	0.48	0.004
mmu-miR-292b-5p	MIMAT0029864	13	5	0.42	-1.25	0.76	0.007
mmu-miR-6991-5p	MIMAT0027884	658	270	0.41	-1.28	0.21	0.021
mmu-miR-7053-5p	MIMAT0028010	3090	1539	0.50	-1.01	0.10	0.039

Supplemental Information 1; Gels and blots of the images Fig. 4e,g

Figure 4e Blots of Bax and β-actin

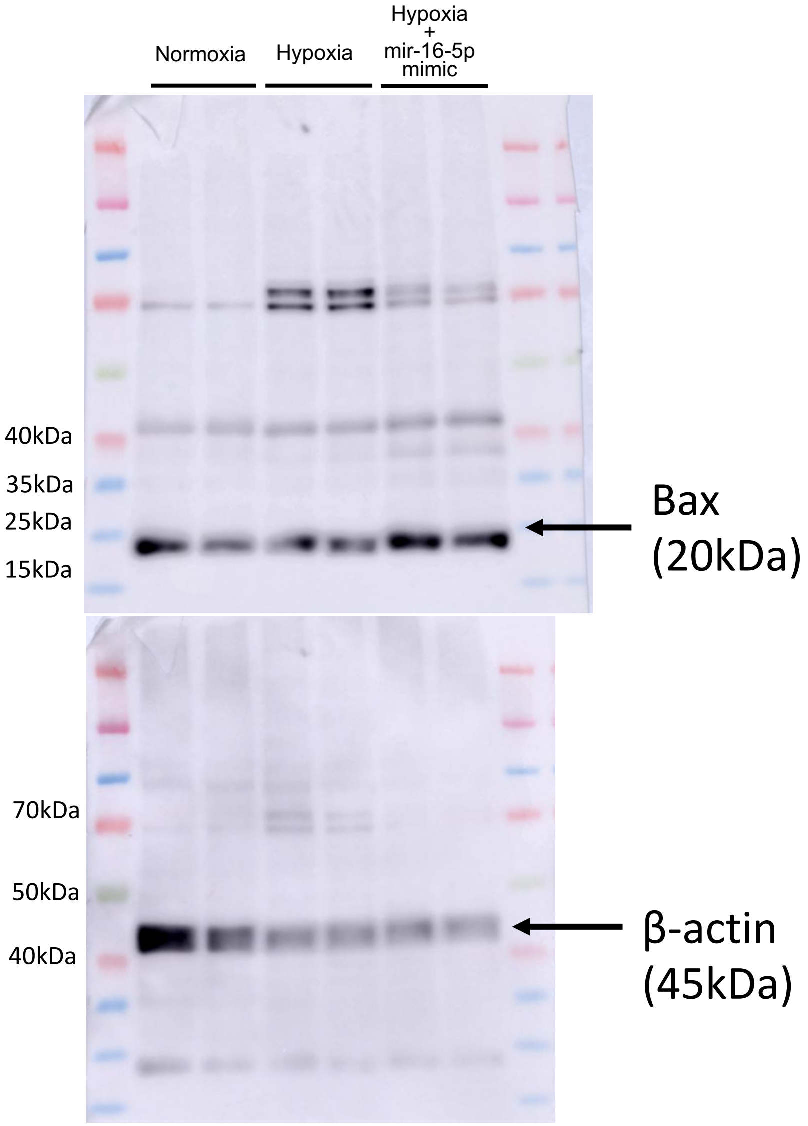

Figure 4g Blots of Pro-/Cleaved-Caspase and β-actin

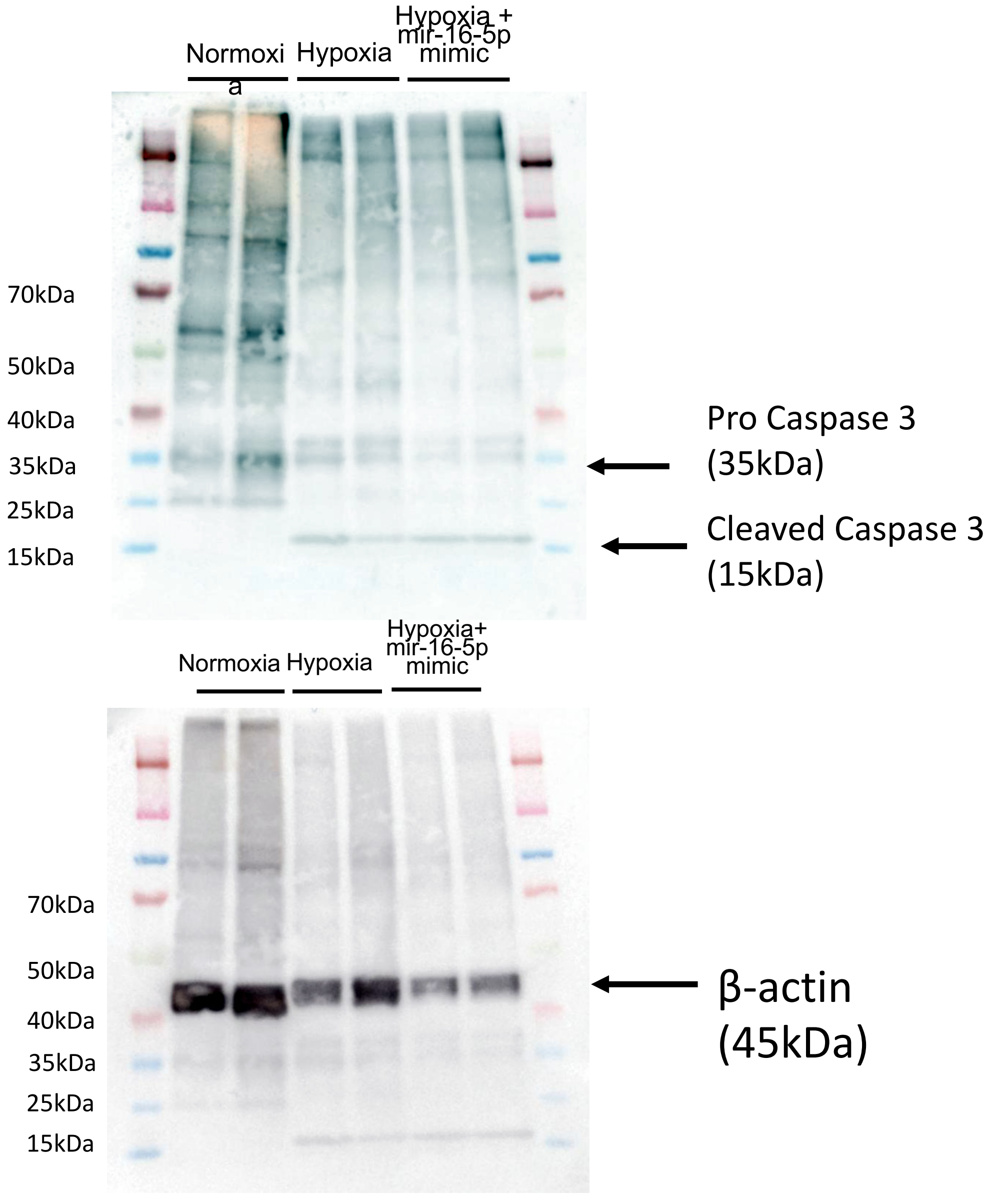

Supplemental Information 2; Gels and blots of the images Fig 5c

Figure 5c Blots of SESN1 and β-actin

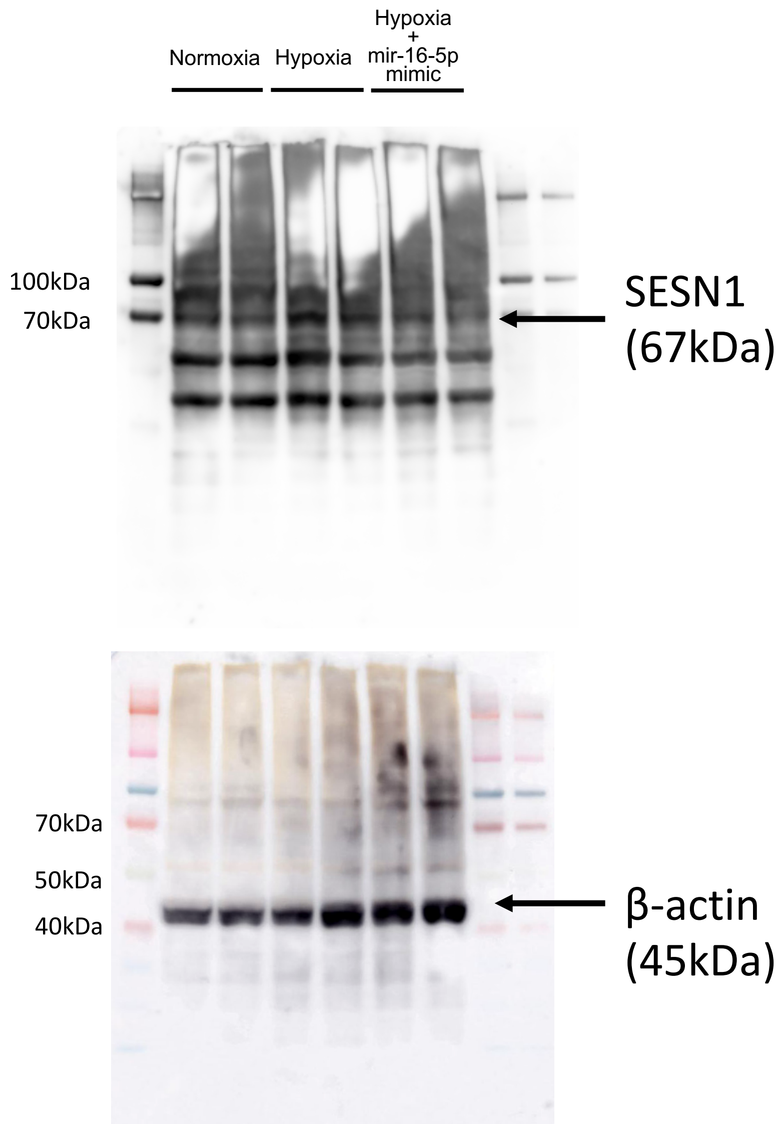

Figure 5c Blots of p-mTOR, mTOR, and β-actin

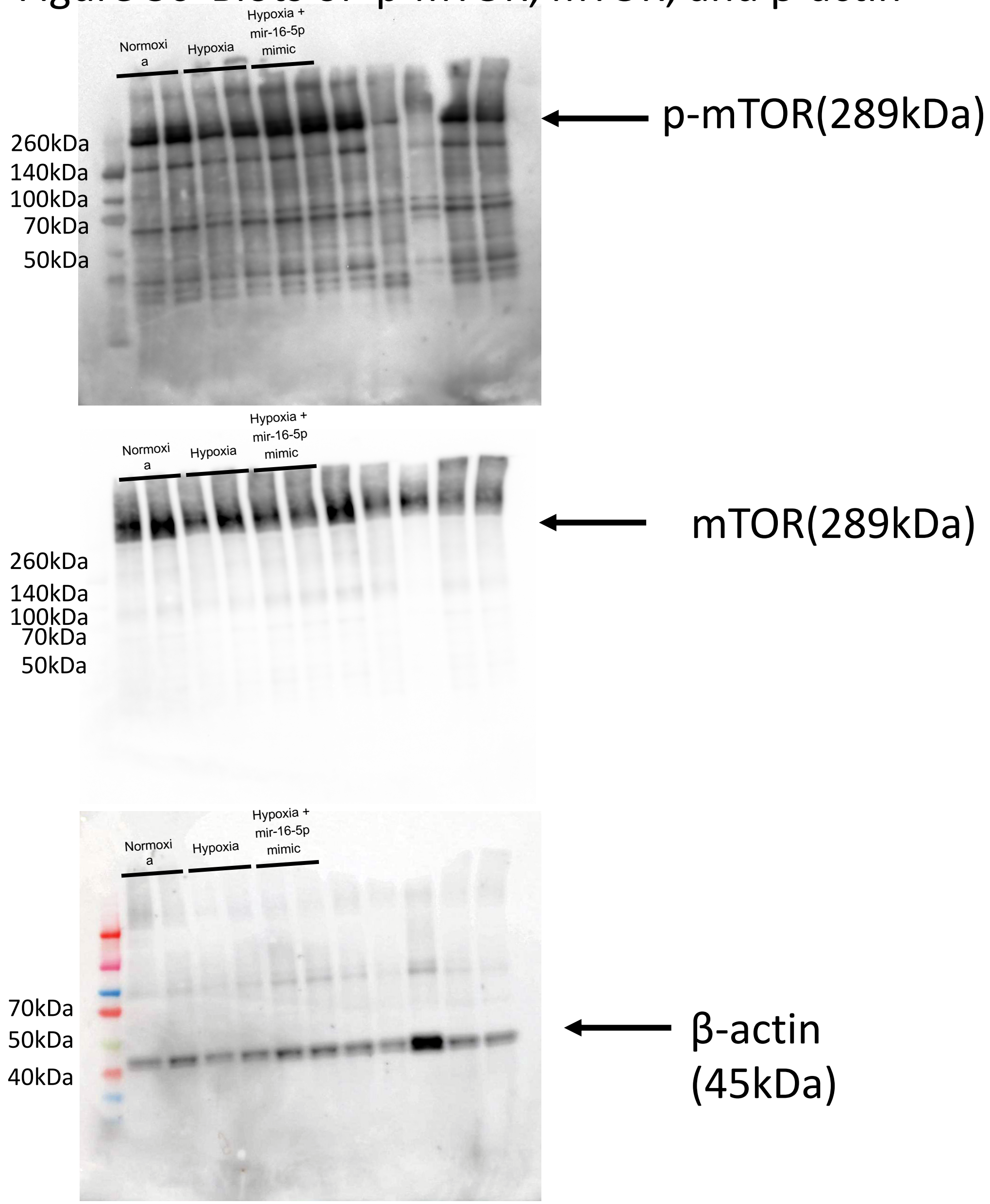

Supplemental Information 3; Gels and blots of the images Fig. 5h

Figure 5h Blots of LCB-II and β-actin

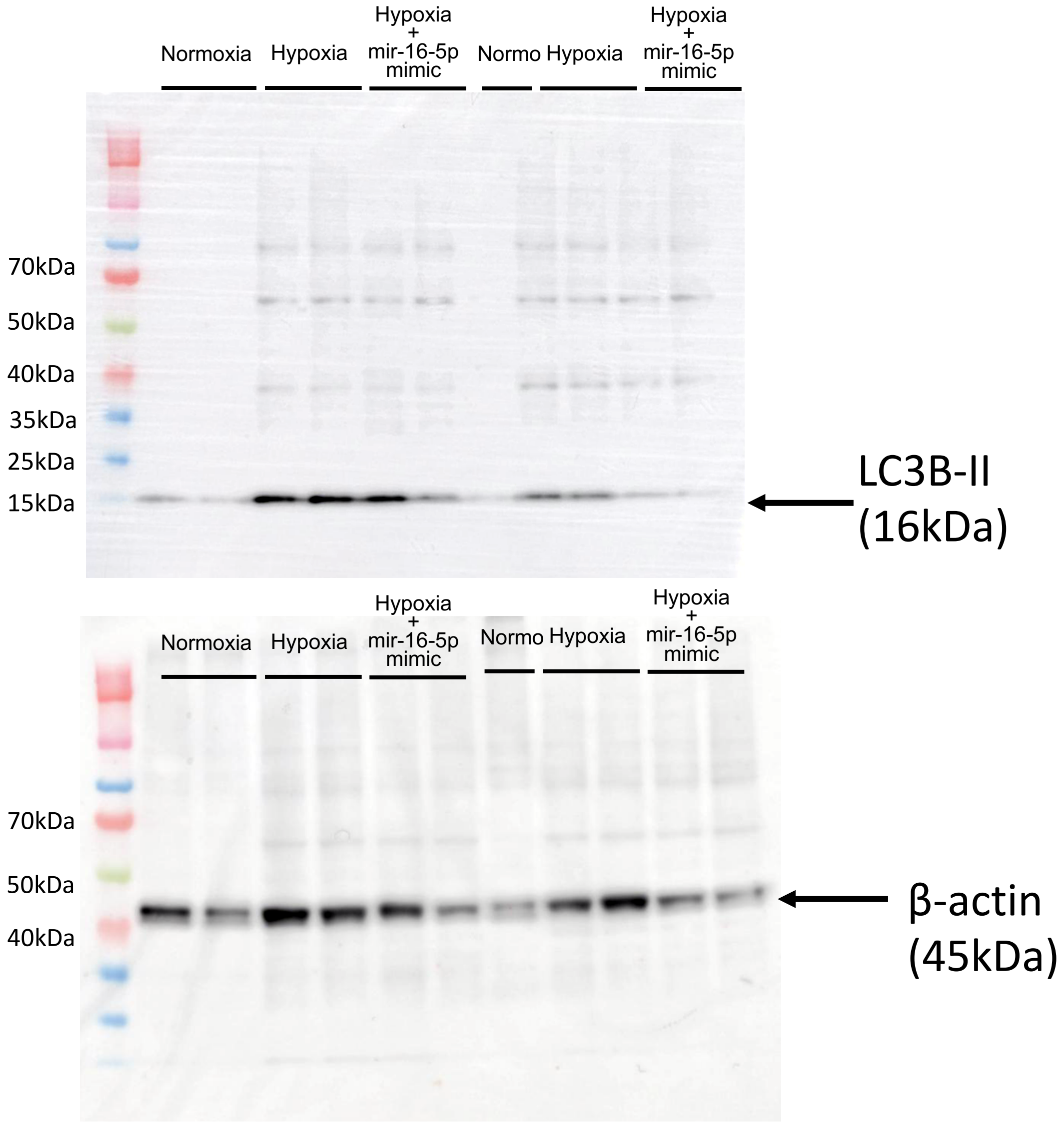

Supplement: Supplementary file 1 — Supplementary Information. [file 41598_2021_98761_MOESM1_ESM.pdf]
